# A Novel Information Complexity Approach to Score Receiver Operating Characteristic (ROC) Curve Modeling

**DOI:** 10.3390/e26110988

**Published:** 2024-11-17

**Authors:** Aylin Gocoglu, Neslihan Demirel, Hamparsum Bozdogan

**Affiliations:** 1 Department of Statistics, The Graduate School of Natural and Applied Sciences, Dokuz Eylul University, Izmir 35390, Turkey; 2 Department of Statistics, Faculty of Science, Dokuz Eylul University, Izmir 35390, Turkey; neslihan.ortabas@deu.edu.tr; 3 Department of Business Analytics and Statistics, University of Tennessee, Knoxville, TN 37996, USA; bozdogan@utk.edu

**Keywords:** Information Complexity, ROC curve, model selection, ICOMP-ROC, Bi-distributional ROC, Universal ROC, genetic algorithm, performance of classifiers

## Abstract

Performance metrics are measures of success or performance that can be used to evaluate how well a model makes accurate predictions or classifications. However, there is no single measure since each performance metric emphasizes a different classification aspect. Model selection procedures based on information criteria offer a quantitative measure that balances model complexity with goodness of fit, providing a better alternative to classical approaches. In this paper, we introduce and develop a novel Information Complexity–Receiver Operating Characteristic, abbreviated as ICOMP-ROC, criterion approach to fit and study the performance of ROC curve models. We construct and derive the Universal ROC (UROC) for a combination of sixteen Bi-distributional ROC models to choose the best Bi-distributional ROC by minimizing the ICOMP-ROC criterion. We conduct large-scale Monte Carlo simulations using the sixteen Bi-distributional ROC models with the Normal–Normal and Weibull–Gamma pairs as the pseudo-true ROC models. We report the frequency of hits of the ICOMP-ROC criterion, showing its remarkable recovery rate. In addition to Bi-distributional fitting, we consider a high-dimensional real Magnetic Resonance Imaging (MRI) of the Brain dataset and Wisconsin Breast Cancer (WBC) dataset to study the performance of the common performance metrics and the ICOMP-ROC criterion using several machine learning (ML) classification algorithms. We use the genetic algorithm (GA) to reduce the dimensions of these two datasets to choose the best subset of the features to study and compare the performance of the newly proposed ICOMP-ROC criterion along with the traditional performance metrics. The choice of a suitable metric is not just contingent upon the ML model used, but it also depends upon the complexity and high dimensionality of the input datasets, since the traditional performance metrics give different results and have inherent limitations. Our numerical results show the consistency and reliability of the ICOMP-ROC criterion over the traditional performance metrics as a clever model selection criterion to choose the best fitting Bi-distributional ROC model and the best classification algorithm among the ones considered. This shows the utility and the versatility of our newly proposed approach in ROC curve modeling that integrates and robustifies currently used procedures.

## 1. Introduction

A Receiver Operating Characteristic (ROC) curve is a unit square plot for simultaneously displaying the tradeoff between the True Positive Rate (TPR), which is the probability that the model correctly predicts the positive class, and the False Positive Rate (FPR), which is the probability that the model incorrectly predicts the positive class, for a binary classifier at different classification thresholds. Therefore, the ROC curve is one of the widely used classification tools that helps in assessing the performance of the diagnostic tests and comparing these tests using intrinsic and accuracy measures, such as sensitivity, specificity, and the Area Under the Curve (AUC).

In the vast literature on ROC, in terms of Bi-distributions, we see that the most commonly used Bi-distributional ROC is the Bi-normal ROC model. The Bi-normal ROC model assumes that the random variables $X_{1}$ and $X_{2}$ are distributed according to normal (Gaussian) distributions with their respective means and variances [1]. Numerous studies have applied the Bi-normal model, including the recent papers in medical research by Shibata et al. [2] and Wei et al. [3]. However, in most scientific fields of investigation, there is no guarantee that the data will follow a normal distribution. Hence, there can be substantial bias in the pointwise estimates of the estimated ROC curve, which in turn can creates inaccurate thresholds for the final decision rule. This situation has created a new opportunity and need for new research direction in ROC models when the normal distributional assumption is not achievable. In terms of other Bi-distributions, some notable studies include Bi-Beta [4], Bi-Exponential [5], Bi-Gamma [6], and Bi-Weibull [7], to mention a few. Despite many years of scientific effort, achieving a comprehensive generalization has remained elusive and an open area of new research work by many others, as briefly reviewed in recent related work (Section 2). For example, for the Bi-distributional ROC curve model, Gneiting and Walz [8] and Gneiting and Vogel [9] proposed the Universal ROC (UROC) curve, which represents a generalized form of ROC curve model that overcomes the extent of the present shortcomings in the literature. The UROC curve combines multiple individual ROC curves into a single curve, weighted based on class configurations. These configurations are determined by the unique values of the outcome. This process is performed in a well-defined manner, ensuring the accurate representation of the overall model performance.

For high-dimensional datasets, the success of statistical modeling techniques depends on identifying and selecting the most informative predictor variables. High-dimensional data often have many redundant variables (or features) and a small number of relevant variables. The presence of redundant variables deteriorates the performance of classification machine learning (ML) algorithms. Therefore, it is crucial to identify and choose the relevant variables when datasets contain numerous explanatory variables and there is limited prior knowledge about their importance. Because of this, choosing the most relevant variables is a challenge for researchers. When the goal is to classify the high-dimensional datasets and to minimize the number of false positives and false negatives while maximizing the number of true positives and true negatives, model selection procedures based on information criteria offer a superior alternative to classical approaches.

Therefore, in summary, our objective and contributions in this paper are several-fold.

- To address and resolve the existing problems in currently practiced ROC curve modeling, for the first time, we introduce and develop a new Information Complexity–Receiver Operating Characteristic (ICOMP-ROC) criterion.
- Using the UROC curve formalism, we generalize the Bi-distributional ROC curve model to the inventory of Bi-distributions and show how to choose the best-fitting ROC curve model with the ICOMP-ROC criterion.
- The performance metrics fall short because they do not simultaneously consider model complexity, especially in complex high-dimensional datasets. To robustify the performance metrics of classifiers in high dimensions we use the genetic algorithm (GA) in selecting the best subset of features with ICOMP-ROC that provides a comprehensive framework for evaluating model performance and complexity at the same time, thereby facilitating more informed, reliable, and interpretable results in the decision-making process.

We organize the rest of the paper as follows. In Section 2, we review the most recent work on ROC models related to our study and their applications. In Section 3, we briefly provide the definition of the Universal ROC Curve (UROC). In Section 4, we discuss the general background of the Information Complexity (ICOMP) criterion. In Section 5, we introduce the new ICOMP-ROC criterion and give its derived form for the bivariate normal (Gaussian) distribution in matrix form. Section 6 presents an inventory of Bi-distributional ROC curve models using different combinations of probability distributions, with the Normal–Normal (BiNormal) and Weibull–Gamma being the pseudo-true pair of Bi-distributional models. The results of large Monte Carlo simulations under two pseudo-true and a large class of symmetric and asymmetric Bi-distributions commonly fitted to real datasets are provided in Section 6.1, along with AUC, AIC-ROC, and ICOMP-ROC values for all the positive and negative classes of distributions. Further, we present the frequency of success for the Monte Carlo simulation studies. Section 7 is devoted to two real numerical examples and presents the results of the analysis conducted on Magnetic Resonance Imaging (MRI) of the Brain data with preprocessing and feature extraction of the Brain data and Wisconsin Breast Cancer data. In Section 7.1, we present and use the genetic algorithm (GA) to select the optimal subset of features for dimension reduction to improve the classification accuracies of the machine learning (ML) classification algorithms. Section 7.2 presents several ML classification algorithms, including logistic regression (LR), support vector machines (SVMs), Naive Bayes (NB), k-nearest neighbor (KNN), and Decision Trees (DT), which are also considered in the recent related work in Section 2. Section 7.3 presents briefly the traditional performance metrics and how our newly proposed ICOMP-ROC model selection criteria are computed in the classification problems. In Section 7.4, we present our main computational results on the two real datasets and compare the performance of our newly proposed ICOMP-ROC model selection criteria with other traditional performance metrics. Finally, Section 8 provides our conclusions and discussion.

## 2. Recent Related Work

In reviewing the vast literature on Receiver Operating Characteristic (ROC) curve modeling, most recently, similar to our proposed approach in this paper, we see a new direction of work by several authors who have studied the utility and performance of the ROC curve model in various application areas.

Pendrill et al. [10] discuss how the popular ROC curves are evaluated with an attempt to modernize the ROC curve with its inherent limitations of classic test theory (CTT) such as non-linearity, the effects of ordinality and confounding task difficulty, and instrument ability. They take an approach of combining Measurement System Analysis (MSA) and Item Response Theory (IRT) and examine ROC curves in explicit terms of the Rasch model. They present a case study in pregnancy testing in order to exemplify the need for improved performance metrics and the establishment of performance goals for devices with binary responses. They advocate the linearisation of the traditional ROC curve.

Reshan et al. [11] use the Wisconsin Breast Cancer (WBC) benchmark dataset and explore automated breast cancer (BC) prediction using multi-model features and ensemble machine learning (EML) techniques. In the feature extraction process, they suggest a Recursive Feature Elimination (RFE) technique to find the most important features of the WBC that are pertinent to BC detection and classification. They propose machine learning (ML) models to obtain high classification accuracy by adapting and combining the EML model for BC diagnosis. The ML models they consider include many well-established classification algorithms to study the performance metrics and to compare their results.

Han [12] presented the results of the performance of the ROC curve in educational assessment and studied the accuracy and consistency of classification results.

Hichri et al. [13] used the genetic algorithm (GA)-based neural network (NN) for fault detection and diagnosis with applications to grid-connected photovoltaic (PV) systems to reduce the number of input features, presenting different scenarios of faults. They used the performance metrics for validation on a grid-connected PV system using a neural network (NN) and a GA-based NN to study the accuracy of fault classification results. To validate the robustness and effectiveness of their method, they proposed other classifiers such as the Recurrent Neural Network (RNN), Long Short-Term Memory (LSTM), Convolution Neural Network (CNN), Feed-Forward Neural Network (FFNN), and Cascade-Forward Neural Network (CFNN).

Ibrahim et al. [14] presented a new hybrid Invasive Weed Optimization (IWO) and machine learning approach for fault detection. They used an IWO-based optimal subset to reduce the data dimension to increase the average accuracy of the model. The optimal subset of features was fed into three well-known classification algorithms, which were trained using k-fold cross-validation to distinguish between the induction motor faults. A similar strategy was performed by applying the genetic algorithm (GA) to compare with the performance of the proposed method. The suggested fault detection model’s performance was evaluated by calculating the ROC curve and the performance metrics. Their experimental results showed the superiority of IWO for selecting the discriminant features, which has achieved more than 99.7% accuracy.

In summary, despite all these most recent advances in ROC curve modeling, still there is a gap in the literature in terms of model selection via the Information Complexity approach, whether it is choosing the best Bi-distributional ROC curve model or the best classification technique among a portfolio of machine learning (ML) classification algorithms. To this end, our proposed new approach, as we listed under our contributions, is a unique contribution to the new direction of research in ROC curve modeling.

## 3. Universal ROC Curve

Consider a pair of random variables, *X* and *Y*, with the joint distribution Q. In this context, *X* represents a real-valued score, while *Y* is a binary event, with the implicit understanding that higher values of *X* indicate stronger support for the event *Y* to occur (Y=1). The joint distribution Q for the pair (X,Y) is defined by the prevalence that falls within the range of [0,1]. Let $\pi_{0}=1-\pi_{1}=Q\left(Y=0\right)$, and let

(1) $$F\left(x\right)=Q\left(X\leq x\right)=\pi_{0}F_{0}\left(x\right)+\pi_{1}F_{1}\left(x\right)$$

denote the marginal cumulative distribution function (cdf) of the score *X*. In addition, this distribution is characterized by conditional cumulative distribution functions (cdf’s) defined by

(2) $$F_{1}\left(x\right)=Q\left(X\leq x|Y=1\right)\;\mathrm{and}\;F_{0}\left(x\right)=Q\left(X\leq x|Y=0\right).$$

Any threshold value *x* can be used to predict a positive outcome (Y=1) if X>x and a negative outcome (Y=0) if X≤x , to yield a classifier with TPR and FPR, as given in Equations (3) and (4), respectively.

(3) $$TPR\left(x\right)=Q\left(X>x|Y=1\right)=1-F_{1}\left(x\right)$$

(4) $$FPR\left(x\right)=Q\left(X>x|Y=0\right)=1-F_{0}\left(x\right)$$

The ROC curve is a representation that is created through the linear interpolation of raw ROC diagnostics. It is also a point set that may admit a direct interpretation as a function. In the case where both $F_{1}$ and $F_{0}$ are continuous and strictly increasing functions, the raw ROC diagnostic and the ROC curve can be identified by a function R, as given in Equation (5):

(5) $$R\left(p\right)=\left\{\begin{array}{cc} 0, & p=0,\\ 1-F_{1}\left(F_{0}^{-1}\left(1-p\right)\right), & p\in (0,1),\\ 1, & p=1. \end{array}\right.$$

In data analytic practice, the measure Q is the empirical distribution of a sample ${\left(x_{i},y_{i}\right)}_{i=1}^{n}$ of real-valued scores $x_{i}$ and corresponding binary observations $y_{i}$. Considering the unique values of $x_{1},\ldots ,x_{n}$ is sufficient for generating the raw ROC diagnostic, and linear interpolation yields the empirical ROC curve [8,9].

## 4. A Brief Background of Information Complexity Criterion

In the literature, the importance of model selection based on information criteria has been recognized and well established by the introduction of the celebrated Akaike’s Information Criterion (AIC) by Akaike [15] as an alternative to classical inferential procedures.

AIC is a criterion based on assessing the model’s lack of fit and penalizing the number of parameters defined by

(6) $$AIC=-2\log L({\hat{\theta }}_{k})+2k$$

where *k* is the number of estimated parameters in the model, ${\hat{\theta }}_{k}$ is the maximum likelihood estimate (MLE) of $\theta_{k}$, and $L({\hat{\theta }}_{k})$ is the maximized likelihood function. The first term in the AIC serves as the lack-of-fit component, and 2k is the penalty term. The model with the minimum AIC value is chosen as the best model to fit the data. Many penalized likelihood-based model selection criteria (AICc, CAIC, CAICF, TIC, etc.) have been developed based on Akaike’s work. See Bozdogan [16].

Later, inspired by Akaike’s AIC, Bozdogan developed the informational complexity (ICOMP) criterion. ICOMP not only considers the goodness of fit and model simplicity but also it takes the complexity of the model into account.

The general formulation of ICOMP is based on the covariance complexity index of van Endem [17] in parametric estimation. Instead of penalizing the number of free parameters directly, the ICOMP penalizes the covariance complexity of the model. Consider a general multivariate linear or nonlinear model defined by

(7) StatisticalModel=Signal+Noise.

ICOMP is designed to estimate the loss function

$$\left.\begin{array}{ccc} \left.\begin{array}{ccccc} & & \;\;Lack\;of\;fit & & \\ & & \;\;Loss=+Lack\;of\;Parsimony & \end{array}\right\}\Rightarrow \;AIC\\ \;\;\;\;+Profusion\;of\;Complexity & & \end{array}\right\}\Rightarrow ICOMP$$

in several ways using the additivity properties of information theory. In AIC, a compromise occurs between the maximized log-likelihood, $-2\log L({\hat{\theta }}_{k})$ (the lack-of-fit component), and *k*, the number of free parameters estimated within the model (the penalty component), which is a measure of complexity that compensates for the bias in the lack of fit when the MLE is used. On the other hand, ICOMP has a third term in the loss function called the ’Profusion of Complexity’, which measures how the parameter estimates are correlated with one another in the model fitting process. Therefore, instead of penalizing the number of free parameters directly, the ICOMP penalizes the covariance complexity of the model. It is defined by

(8) $$\;ICOMP=-2\log L\left({\hat{\theta }}_{k}\right)+2C\left({\hat{\Sigma }}_{model}\right)\;$$

where *L* is the likelihood function, ${\hat{\theta }}_{k}$ is an estimator of the unknown parameter $\theta_{k}$, *C* represents a real-valued complexity measure; $\hat{Cov}\left({\hat{\theta }}_{k}\right)={\hat{\Sigma }}_{model}$ represents the estimated covariance matrix of the parameter vector of the model. The most general form of ICOMP, called ICOMP (IFIM) based on Equation (9), takes advantage of the well-known asymptotic optimality properties of MLEs and uses the estimated inverse Fisher information matrix (IFIM) to measure the complexity of a model. In this case, the most general form of the ICOMP is given by

(9) $$\;ICOMP\left(IFIM\right)=-2\log\left(\hat{\theta }\right)+2C_{1}\left({\hat{F}}^{-1}\right),\;$$

where $C_{1}$ denotes the maximal Information Complexity of $\left({\hat{F}}^{-1}\right)$ given by

(10) $$\;C_{1}\left({\hat{F}}^{-1}\right)=\frac{s}{2}\log L\left(\frac{tr({\hat{F}}^{-1})}{s},\right)-\frac{1}{2}\log\left|{\hat{F}}^{-1}\right|,\;$$

where ${\hat{F}}^{-1}=\hat{Cov}\left({\hat{\theta }}_{k}\right)$, $s=dim\left({\hat{F}}^{-1}\right)=rank\left({\hat{F}}^{-1}\right)$, $tr({\hat{F}}^{-1})$ denotes the trace of IFIM which measures the average total variation, and $\left|{\hat{F}}^{-1}\right|$ denotes the determinant of IFIM, which measures the generalized variance. In this way, entropic complexity combines the two measures of variations in a high-dimensional dataset. For more details on this clever criterion and its other general forms, we refer the readers to Bozdogan [18] and Sun and Bozdogan [19].

## 5. A Newly Proposed ICOMP-ROC Criterion

To define the ICOMP-ROC criterion, let $X_{1}$ and $X_{2}$ denote *False Positive Rate* (*FPR*) and *True Positive Rate* (*TPR*). Let $\left(X_{1},X_{2}\right)$ have the bivariate normal (Gaussian) distribution with the joint probability density function given by

(11) $$\begin{array}{cc} f_{X_{1}X_{2}}\left(x_{1},x_{2}\right) & =\frac{1}{2\pi \sigma_{1}\sigma_{2}{\left(1-\rho \right)}^{1/2}} \end{array}$$

(12) $$\begin{array}{cc} \; & \times exp\left\{-\frac{1}{2{\left(1-\rho \right)}^{2}}\left[{\left(\frac{x_{1}-\mu_{1}}{\sigma_{1}}\right)}^{2}-2\rho \left(\frac{x_{1}-\mu_{1}}{\sigma_{1}}\right)\left(\frac{x_{2}-\mu_{2}}{\sigma_{2}}\right)+{\left(\frac{x_{2}-\mu_{2}}{\sigma_{2}}\right)}^{2}\right]\right\} \end{array}$$

where ρ is the correlation coefficient between $X_{1}$ and $X_{2}$. It is given by

(13) $$\rho =\frac{Cov(x_{1},x_{2})}{\sigma_{1}\sigma_{2}}.$$

In matrix notation, we denote the bivariate normal distribution as

(14) $$\left(\begin{array}{c} X_{1}\\ X_{2} \end{array}\right)∼N_{p=2}\left(\mu =\left[\begin{array}{c} \mu_{1}\\ \mu_{2} \end{array}\right],\;\Sigma =\left[\begin{array}{cc} \sigma_{1}^{2} & \rho \sigma_{1}\sigma_{2}\\ \rho \sigma_{1}\sigma_{2} & \sigma_{2}^{2} \end{array}\right]\right).$$

This pdf is often used in many applications to model the joint pdf of two random variables $X_{1}$ and $X_{2}$. It has five parameters $\theta =\left\{\mu_{1},\mu_{2},\sigma_{1}^{2},\sigma_{2}^{2},\rho \right\}.$

The *standard bivariate normal distribution* in terms of the *sample correlation matrix R* is denoted as

(15) $$\left(\begin{array}{c} X_{1}\\ X_{2} \end{array}\right)∼N_{p=2}\left(\mu =\left[\begin{array}{c} 0\\ 0 \end{array}\right],R=\left[\begin{array}{cc} 1 & r\\ r & 1 \end{array}\right]\right).$$

In general, the probability density function (pdf) of the p-dimensional multivariate normal (or Gaussian) distribution is

(16) $$f\left(x,\mu ,\Sigma \right)=\frac{1}{\sqrt{{\left(2\pi \right)}^{p}|\Sigma |}}\exp\left(-\frac{1}{2}\left(x-\mu \right)\Sigma^{-1}{\left(x-\mu \right)}^{\prime }\right),$$

where *x* and μ are (1×p) vectors and Σ is a (p×p) symmetric, positive definite matrix.

Under the bivariate normal, in terms of the correlation matrix *R*, the *estimated inverse Fisher information matrix* (*IFIM*), after some work, is given by

(17) $${\hat{F}}^{-1}=\left[\begin{array}{cc} R & 0\\ 0 & \;\;\frac{2}{n}D_{2}^{+}\left(R⊗R\right)D_{2}^{{+}^{\prime }} \end{array}\right]$$

where $D_{p}^{+}={\left(D_{p}^{\prime }D_{p}\right)}^{-1}D_{p}^{\prime }$ is the Moore–Penrose inverse of the duplication matrix. The duplication matrix is a unique $p^{2}\times \frac{1}{2}p\left(p+1\right)$ matrix that transforms, for symmetric matrix *A*, vech(A) into $vec\left(A\right)$. That is,

(18) $$D_{p}vech\left(A\right)=vec\left(A\right)\;\left(A=A^{\prime }\right),$$

where $vech\left(A\right)$ denotes the $\frac{1}{2}p\left(p+1\right)\times 1$ vector that is obtained from $vec\left(A\right)$ by eliminating all subradiagonal elements of A. For example, for *p* = 2; that is, for a (2×2) symmetric matrix *A*, we have

(19) $$A=\left(\begin{array}{cc} a_{11} & a_{12}\\ a_{21} & a_{22} \end{array}\right)\;vec\left(A\right)={\left(a_{11},a_{21},a_{12},a_{22}\right)}^{\prime }\;and\;vech\left(A\right)={\left(a_{11},a_{21},a_{22}\right)}^{\prime },$$

where the supradiagonal element $a_{12}$ has been removed. Then,

(20) $$D_{2}vech\left(A\right)=\left(\begin{array}{ccc} 1 & 0 & 0\\ 0 & 1 & 0\\ 0 & 1 & 0\\ 0 & 0 & 1 \end{array}\right)\left(\begin{array}{c} a_{11}\\ a_{21}\\ a_{22} \end{array}\right)=\left(\begin{array}{c} a_{11}\\ a_{21}\\ a_{21}\\ a_{22} \end{array}\right)=vec\left(A\right).$$

The duplication matrix improves the computational time of very large estimated inverse Fisher information matrix (IFIM) in many applications.

Using the definition of ICOMP in Equation (9), based on IFIM, we now define the ICOMP-ROC criterion as

(21) $$ICOMP-ROC\left({\hat{F}}^{-1}\right)=-2logL\left(\hat{\theta }\right)+2C_{1}\left({\hat{F}}^{-1}\right),$$

and derive its analytical form for the ROC curve model as our fitness function given by

(22) $$ICOMP-ROC\left({\hat{F}}^{-1}\right)=n\left(log(2\pi )+log|R|+1\right)+2C_{1}\left({\hat{F}}^{-1}\right),$$

where $n=n_{1}+n_{2}$ is the total sample size. Note that the sample sizes do not need to be equal. Further,

(23) $$C_{1}\left({\hat{F}}^{-1}\right)=\frac{s}{2}\log\left(\frac{tr({\hat{F}}^{-1})}{s}\right)-\frac{1}{2}\log\left|{\hat{F}}^{-1}\right|\;$$

is the maximal entropic complexity of ${\hat{F}}^{-1}$, *IFIM* of the bivariate normal (Gaussian) model.

For the bivariate case, for *p* = 2, the opened-up form of $C_{1}\left({\hat{F}}^{-1}\right)$ in terms of the correlation matrix *R* is obtained as

(24) $$C_{1}\left({\hat{F}}^{-1}\right)=\frac{s}{2}log\left(\frac{tr\left(R\right)+\frac{1}{n}\left[tr\left(R\right)+{\left(tr(R)\right)}^{2}+4\right]}{s}\right)-2log\left|R\right|+\frac{3}{2}log\left(n\right)-log\left(2\right),$$

where $s=rank\left({\hat{F}}^{-1}\right)$. Note that by computing the complexity in this way, we avoid building the large IFIM, and we only need traces and determinants of IFIM, which is computationally efficient.

AIC-ROC is defined by

(25) $$AIC-ROC=n\left(log(2\pi )+log|R|+1\right)+2rank\left(R\right).$$

The rationale for proposing bivariate normal (Gaussian) distribution to fit and score the ROC curve models stems from the fact that FPR and TPR are dependent, rather than being independent.

Similar to the interpretation of AIC and ICOMP values, the best result is determined by the minimum value of AIC-ROC and ICOMP-ROC. Lower values of these criteria indicate a better model fit and a more accurate identification of the true distribution. This follows the general principle in model selection, where minimizing AIC or ICOMP reflects an optimal balance between model complexity and goodness of fit, as commonly discussed in the literature [16,18]. Applying this concept to ROC analysis ensures that the models with the lowest AIC-ROC and ICOMP-ROC values are considered the most suitable.

## 6. Large Scale Monte Carlo Simulation Studies

In this section, we present the large-scale Monte Carlo simulations that empirically compare the AIC-ROC and ICOMP-ROC criteria with the widely known AUC from the literature. The aim of these simulations is to demonstrate that when the distributions of the negative class $\left(X_{1}\right)$ and positive class $\left(X_{2}\right)$ are unknown, the ICOMP-ROC criterion is more effective at identifying the correct distribution compared to AUC. For this purpose, we assume that the distributions of $X_{1}$ and $X_{2}$ are derived from Normal–Normal and Weibull–Gamma distributions, respectively, with sample sizes of $n_{X_{1}}=200$ and $n_{X_{2}}=300$. Then, sixteen different Bi-distribution combination scenarios are constructed by using the probability distributions Normal, Exponential, Weibull, Gamma, EV, GEV and GP for the negative class $X_{1}$ and positive class $X_{2}$ . Table 1 represents several of these distributions. Table 2 presents the cumulative distribution functions (cdf’s), and Table 3 presents the inverse cumulative distributions (icdf’s) of the probability distributions in Table 1.

These cdf’s and icdf’s are then used to derive the Universal ROC Curve (UROC) in Equation (5) by pairing combinations of each of these distributions. For illustration, the Weibull–Gamma pair as our pseudo-true distribution is obtained by Equation (26) for Monte Carlo simulation.

(26) $$R_{WG}\left(p\right)=1-F_{W}\left(\left(F_{G}^{-1}\left(1-p,{\hat{\alpha }}_{G},{\hat{\beta }}_{G}\right)\right),{\hat{\alpha }}_{W},{\hat{\beta }}_{W}\right),$$

where $F_{W}$ is the cumulative distribution function (cdf) of the Weibull probability density function (pdf) and $F_{G}^{-1}$ is the inverse cumulative distribution function (icdf) of the Gamma pdf.

Let $\left({\hat{\alpha }}_{W},{\hat{\beta }}_{W}\right)$ and $\left({\hat{\alpha }}_{G},{\hat{\beta }}_{G}\right)$ denote the maximum likelihood estimators (MLEs) of the Weibull and Gamma distribution, respectively. The MLEs of $\beta_{W}$ and $\alpha_{W}$ for the Weibull distribution are the solutions of the simultaneous equation:

(27) $$\begin{array}{cc} {\hat{\beta }}_{W} & ={\left[\frac{1}{n}\sum_{i=1}^{n}x_{i}^{{\hat{\alpha }}_{W}}\right]}^{1/{\hat{\alpha }}_{W}} \end{array}$$

(28) $$\begin{array}{cc} {\hat{\alpha }}_{W} & =\frac{n}{n\log{\hat{\beta }}_{W}+{\left(\frac{1}{{\hat{\beta }}_{W}}\right)}^{{\hat{\alpha }}_{W}}\sum_{i=1}^{n}x_{i}^{{\hat{\alpha }}_{W}}\log\left(\frac{x_{i}}{{\hat{\beta }}_{W}}\right)-\sum_{i=1}^{n}\log x_{i}}. \end{array}$$

Similarly, for the Gamma distribution, using the log-likelihood function

(29) $$logL\left(\alpha_{G},\beta_{G}|x\right)=n\left(\alpha_{G}log\beta_{G}-log\Gamma \left(\alpha_{G}\right)\right)-\left(\alpha_{G}-1\right)\sum_{i=1}^{n}logx_{i}-\beta_{G}\sum_{i=1}^{n}x_{i}$$

we obtain the MLEs by solving the following equations:

(30) $$\begin{array}{cc} \frac{\partial logL}{\partial \alpha_{G}} & =n\left(log\beta_{G}-\frac{d}{d\alpha_{G}}log\Gamma \left(\alpha_{G}\right)\right)+\sum_{i=1}^{n}logx_{i}=0 \end{array}$$

(31) $$\begin{array}{cc} \frac{\partial logL}{\partial \beta_{G}} & =n\frac{\alpha_{G}}{\beta_{G}}-\sum_{i=1}^{n}x_{i}=0. \end{array}$$

From the second equation, we obtain $\bar{x}=\frac{\alpha_{G}}{\beta_{G}}$. Substituting $\beta_{G}=\frac{\alpha_{G}}{\bar{x}}$ into the first equation, we have

(32) $$n\left(\alpha_{G}-log\bar{x}-\frac{d}{d\alpha_{G}}log\Gamma \left(\alpha_{G}\right)+log\bar{x}\right)=0$$

where $\frac{d}{d\alpha_{G}}log\Gamma \left(\alpha_{G}\right)=\psi \left(\alpha_{G}\right)$ is known as the digamma function. Using Equation (32), we obtain the MLE of $\alpha_{G}$.

From the above equations, we note that for the Weibull–Gamma pair, the maximum likelihood estimators (MLEs) cannot be obtained in a closed analytical form. In such cases, to find the MLEs, we use numerical optimization algorithms. These algorithms begin by assuming starting initial values for the unknown parameters and then proceed iteratively until a convergence or stopping criterion is satisfied.

The estimation of the Area Under the Curve (AUC) is inherently dependent upon the estimation of the ROC curve. The AUC is

(33) $$\begin{array}{cc} AUC & =\int_{0}^{1}R_{WG}\left(p\right)dp \end{array}$$

(34) $$\begin{array}{cc} \; & =\int_{0}^{1}1-F_{W}\left(\left(F_{G}^{-1}\left(1-p,{\hat{\alpha }}_{G},{\hat{\beta }}_{G}\right)\right),{\hat{\alpha }}_{W},{\hat{\beta }}_{W}\right)dp. \end{array}$$

In general, for Bi-distributions this integral numerically is evaluated using the Gauss–Kronrod Quadrature (GKQ) algorithm. See Calvetti et al. [20].

In the final step of the simulation, ICOMP-ROC and AIC-ROC values are computed for the Weibull and Gamma distributions by using Equations (21) and (25), respectively. These steps are then repeated for each of the sixteen Bi-distribution pairs, with the criteria recalculated for every pair.

### 6.1. Results of Monte Carlo Simulation Study

#### 6.1.1. Case 1: Normal–Normal Bi-Distribution Pair

**Figure 1:**
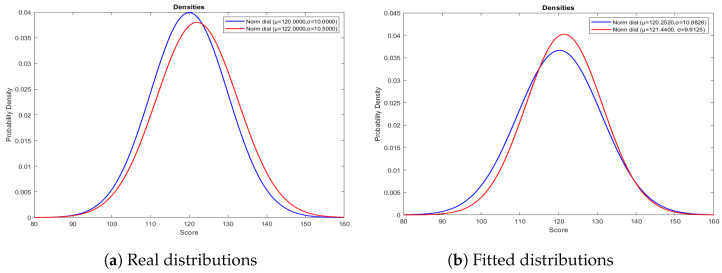
Demonstration of real (**a**) and fitted (**b**) negative and positive classes for the Normal–Normal Bi-distribution pair.

Figure 1a and Figure 1b represent the real and fitted distribution of Normal–Normal pair as our pseudo-true distribution, respectively.

In the simulation study for pseudo-true Normal–Normal distribution pair, Table 4 displays the AUC, AIC-ROC, and ICOMP-ROC values of sixteen different Bi-distribution combination scenarios, covering both symmetric and asymmetric cases commonly encountered in real-world data. Bold values indicate the best result for AUC, AIC-ROC, and ICOMP-ROC. In Table 4, the highest AUC value is obtained for the GP–Normal distribution pair, while the lowest AIC-ROC value is observed for the Exponential–Exponential distribution pair, and the lowest ICOMP-ROC value is achieved for the Normal–Normal distribution pair. As a result, the ICOMP-ROC criterion accurately identifies the correct distribution. In contrast, both AIC-ROC and AUC misidentify the distribution. Furthermore, it is important to emphasize that AUC lacks the information-theoretic foundations provided by AIC-ROC and ICOMP-ROC and that AIC-ROC itself does not account for model complexity as robustly as ICOMP-ROC.

**Figure 2:**
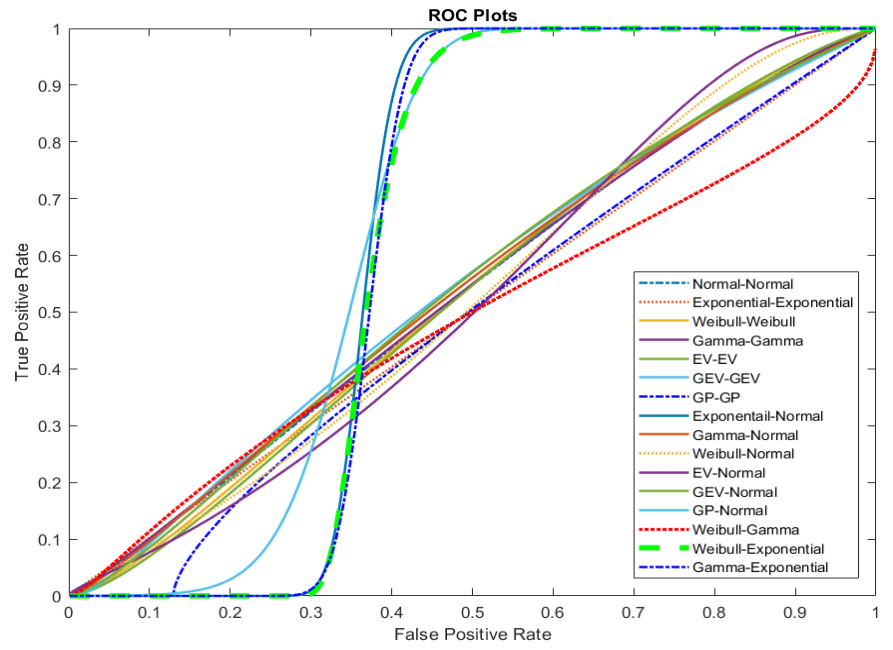
The ROC curves for the sixteen Bi-distribution pairs for pseudo-true Normal–Normal Bi-distribution pair.

The ROC curves for the sixteen Bi-distribution pairs are presented in Figure 2. A curve that is closer to the top-left corner represents a better classifier. According to Figure 2, the GP–Normal distribution pair is closest to the top-left corner. However, the real distribution pair could not be reliably identified.

Table 5 illustrates the frequency of success for the Monte Carlo simulation study across different distribution pairs, comparing the performance of AUC, AIC-ROC, and ICOMP-ROC for 100 runs. Bold values indicate the best result for AUC, AIC-ROC, and ICOMP-ROC. The results indicate that the Normal–Normal Bi-distribution pair achieves a 100% success rate using ICOMP-ROC, correctly identifying the true distribution. In contrast, AUC identifies the GP–Normal distribution pair and AIC-ROC identifies the Exponential–Exponential distribution pair as the best fit, with a 100% success rate in this scenario, but fails to recognize the correct distribution for Normal–Normal. As a result, this further supports the notion that ICOMP-ROC criteria are more reliable and consistent in identifying the real distribution pair, particularly when accounting for information criteria.

#### 6.1.2. Case 2: Weibull–Gamma Bi-Distribution Pair

**Figure 3:**
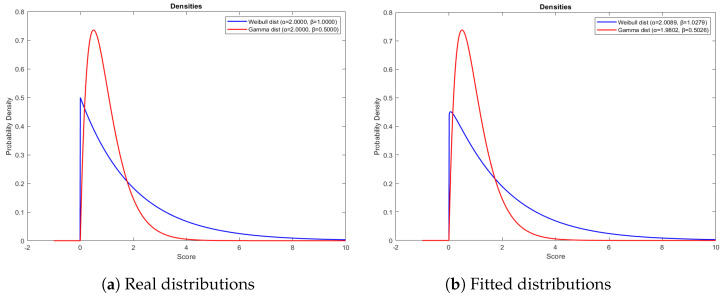
Demonstration of real (**a**) and fitted (**b**) negative and positive classes for the Weibull–Gamma Bi-distribution pair.

Figure 3a and Figure 3b represent the real and fitted distribution of Weibull–Gamma pair as our pseudo-true distribution, respectively.

In the simulation study for pseudo-true Weibull–Gamma distribution pair, Table 6 displays that the best AUC value is obtained for the Weibull-Exponential distribution, while the lowest ICOMP-ROC and AIC-ROC values are achieved for the Weibull–Gamma distribution with bold values indicating the best results for AUC, AIC-ROC, and ICOMP-ROC. As a result, the ICOMP-ROC and AIC-ROC criteria correctly identify this distribution. In contrast, the AUC incorrectly predicts the distribution. Moreover, it is essential to highlight that the AUC lacks the information criteria foundations provided by AIC-ROC and ICOMP-ROC.

**Figure 4:**
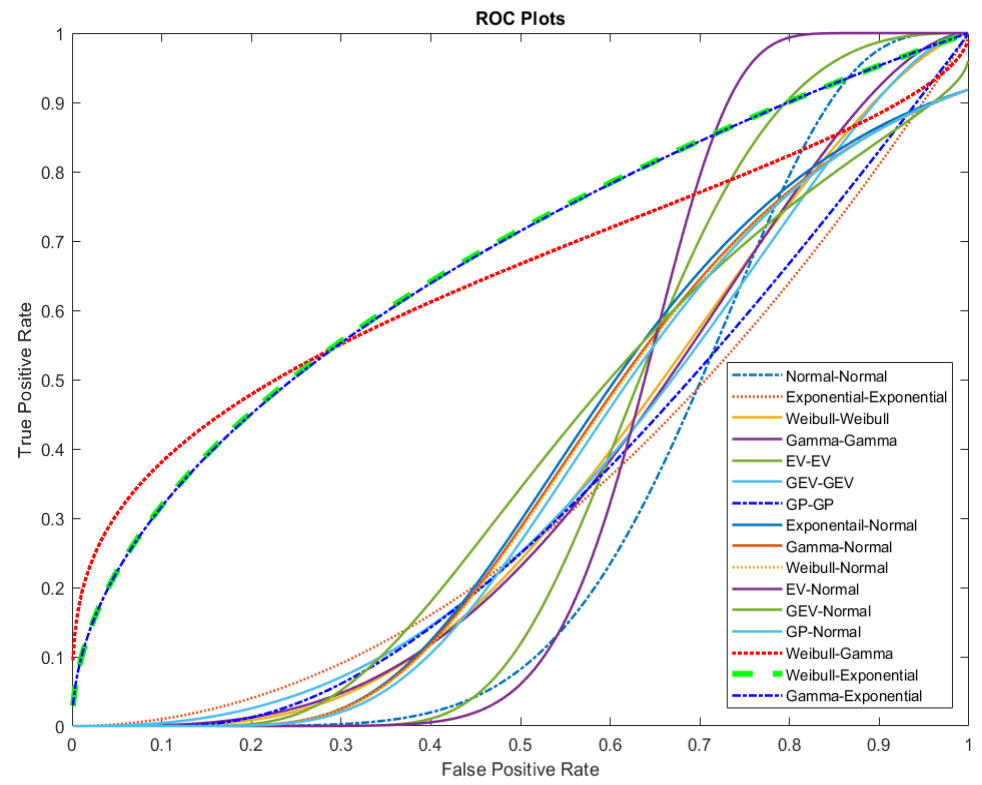
The ROC curves for the sixteen Bi-distribution pairs for the pseudo-true Weibull–Gamma Bi-distribution pair.

According to Figure 4, the Weibull–Exponential and Gamma–Exponential distribution pairs are closest to the top-left corner and exhibit very close ROC curves. However, the real distribution pair could not be reliably identified.

Table 7 illustrates the frequency of success for the Monte Carlo simulation study across different distribution pairs, comparing the performance of AUC, AIC-ROC, and ICOMP-ROC for 100 runs. Bold values indicate the best result for AUC, AIC-ROC, and ICOMP-ROC. The results indicate that the Weibull–Gamma distribution pair achieves a 100% success rate using both AIC-ROC and ICOMP-ROC, correctly identifying the true distribution. In contrast, AUC identifies the Weibull–Exponential distribution as the best fit, with a 100% success rate in this scenario, but fails to recognize the correct distribution for Weibull–Gamma. As a result, this further supports the notion that the AIC-ROC and ICOMP-ROC criteria are more reliable and consistent in identifying the real distribution pair, particularly when accounting for information criteria.

## 7. Real Numerical Examples to Study the Performance of ML Classification Algorithms

In this section, we study the performance of newly proposed information-based criteria, AIC-ROC and ICOMP-ROC, on two real datasets, along with other traditional classification performance metrics.

**Figure 5:**
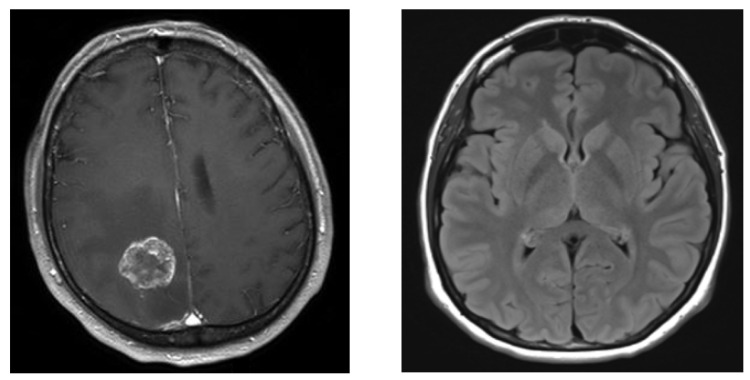
MR images of two classes: malignant (**left**) and benign (**right**) tumors.

**Example 1**: In this example, MRI Brain data were used from the Kaggle repository [21]. A brain tumor is an abnormal growth of cells in the brain. These tumors can be categorized as either benign, which means they are noncancerous and typically grow slowly, or malignant, which means they are cancerous, grow rapidly, and can invade surrounding tissues. The dataset contains 253 MRI images with 155 malignant and 98 benign binary classes. Figure 5 displays the MRI images of the malignant and benign cases.

*Preprocessing*: Data augmentation was applied to increase the diversity of a dataset by applying various transformations to the existing data, such as rotating, scaling, and flipping. A Gaussian filter was used to improve the quality of the image through noise suppression, contract enhancement, intensity equalization, and outlier elimination.

*Feature extraction*: Feature extraction is the procedure of data reduction to find a subset of relevant features based on the image. The gray-level co-occurrence matrix (GLCM) is a statistical method of examining texture that considers the spatial relationship of pixels and is widely used in various image processing applications to quantify different aspects of texture. In this study, the perimeter, area, aspect ratio, bounding rectangle width, bounding rectangle height, energy, correlation, dissimilarity, homogeneity, contrast, and entropy features were extracted from each image. Additionally, the sobel versions of some features (Energy-sobel, Correlation-sobel, Dissimilarity-sobel, Homogeneity-sobel, Contrast-sobel, Entropy-sobel) were derived. As a result, 17 features are extracted based on the GLCM.

**Example 2**: In this example, we consider the Wisconsin Breast Cancer (WBC) data also from the Kaggle repository [22]. These data are widely used to classify breast tumors as benign or malignant for machine learning tasks. It contains 569 instances with 30 numeric features. The target variable indicates the diagnosis.

**Figure 6:**
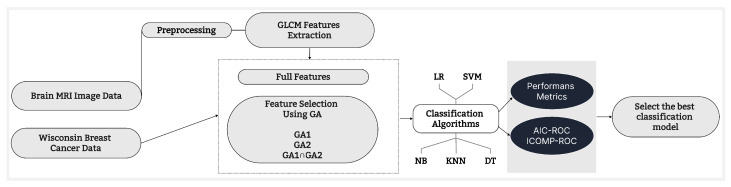
Flowchart of the process.

Figure 6 shows the process steps of the flowchart of our study of the two real datasets.

### 7.1. Feature Selection with the Genetic Algorithm

Since the MRI Brain data consist of images, preprocessing and feature extraction were performed using GLCM (Gray Level Co-occurrence Matrix). For the Wisconsin Breast Cancer dataset, preprocessing is not required and the main features are used directly in the analysis.

For both datasets, the GA is used on all the features using two different GA methods, namely GA1 and GA2, to select the best subset of the features to improve the performance of ML classification algorithms. In addition, the best subset of the overlap features were obtained from both GAs (i.e., GA1 ∩ GA2) by allowing for consistency in the evaluation process and enabling a direct comparison of the effectiveness of the ML classification algorithms under identical conditions. The difference between G1 and G2 approaches is that G2 improves the computational effort by dynamically changing the crossover scheme in each generation of the GA.

In the increasingly important case of high-dimensional datasets, a genetic algorithm (GA) can be used to select the best subset of features. As is well known, GA is a part of evolutionary algorithms inspired by natural selection and genetic operators. GA, a class of evolutionary algorithms, was originally developed by Holland [23]. In addition to offering different approaches to solving problems and consistently outperforming other methods used in searching highly nonlinear spaces in terms of speed and efficiency. GA begins with a population, which is a set of solutions. In GA, each solution is represented by a binary string called a chromosome, which has two possible values: 0 and 1. This will allow inheritance, mutation, and crossover to prevent the local minimum. The criterion to rank solutions is often called a fitness function, and the best-fitting solutions are kept to create the following generation. The retained good solutions will be created when the good solutions that were kept mate. This process is repeated until a specific convergence criterion is reached or optimal solutions are found. The natural selection approach is frequently employed. The chance that an individual will be chosen under this strategy is proportional to the ratio.

Many challenges across data science and machine learning (ML) problems can be solved through the application of GAs. Specifically, the genetic algorithm with ICOMP as the objective function has been successfully used in several research problems by the third author and his doctoral students.

The GA parameters used in this study for G1 and G2 methods are given in Table 8 below to select the best subset of features to improve the performance of ML classification algorithms.

### 7.2. Machine Learning (ML) Classification Algorithms

In this study, we employ fundamental classification algorithms including logistic regression (LR), support vector machine (SVM), Naive Bayes (NB), k-nearest neighbor (KNN), and Decision Tree (DT). A brief overview of these algorithms is as follows.

The LR model is a statistical modeling technique used to predict the probability of a dichotomous variable (e.g., 0 for the event not to occur or 1 for the event to happen) based on one or more input variables. It works by modeling the relationship between variables using a sigmoid function and a decision threshold for classification. SVM is a supervised learning algorithm that finds an optimal hyperplane that maximally discriminates between various classes in an N-dimensional space. The hyperplane is described as a decision boundary that separates the two classes. The data points closest to the decision boundary, called support vectors, have the greatest influence on determining the location of the hyperplane. After identifying the hyperplane, new data are classified based on which side of the hyperplane they fall on. The NB classification algorithm is a simple yet powerful probabilistic classifier based on Bayes’s theorem with the naive feature independence assumption. The KNN algorithm is a nonparametric method used in classification and regression. The working principle of this method is the assignment of data that are new in a previously created sample set to the cluster that has the closest (k) distance. The DT classification is used to categorize data into distinct classes. The method constructs a tree-like model of decisions, where each node represents a feature, each branch denotes the outcome of a decision, and each leaf node assigns a class label. This technique is visually intuitive, making it easy to understand and interpret. For a detailed explanation of the algorithms, see James et al. [24].

### 7.3. Performance Metrics and Information-Based Criteria for Classification

**Figure 7:**
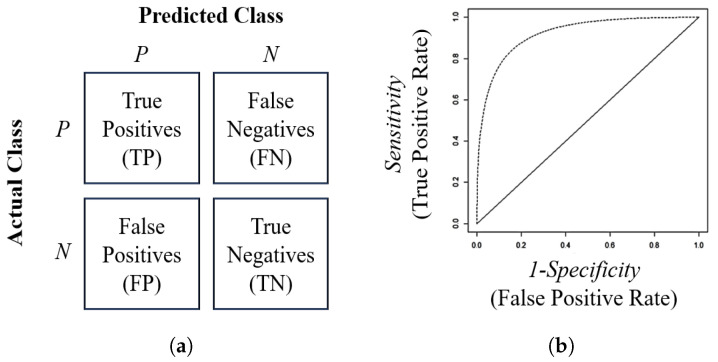
Illustration of a confusion matrix (**a**) and ROC curve (**b**).

A confusion matrix is a table that is often used to evaluate the performance of a classification model. It shows the number of correct and incorrect predictions made by the model compared with the actual outcomes in a test dataset. The rows represent the actual classes, and the columns represent the predicted test results in Figure 7a. Possibilities after cross-classification then include a true positive (TP), which occurs when the model correctly predicts the positive class, and a true negative (TN), which is when the model correctly predicts the negative class. Conversely, a false positive (FP) arises when the model incorrectly predicts the positive class, and a false negative (FN) occurs when the model incorrectly predicts the negative class. Using the confusion matrix, which tabulates the model’s predictions against actual class labels, several performance metrics can be derived. These metrics include accuracy, precision, recall (also known as sensitivity), F1 score, and error rate. Table 9 presents these metrics, which are valuable indicators of a classification model’s effectiveness in accurately classifying instances.

Figure 7b shows the ROC curve, which is a graphical display of sensitivity (True Positive Rate) on the y-axis and 1—specificity (False Positive Rate) on the x-axis for varying cut-off points of test values. The overall performance of a classifier, summarized over all possible thresholds, is given by the area under the ROC curve (AUC). The AUC ranges from 0 to 1, with 0.5 representing a random classifier and 1 representing a perfect classifier [25]. In all performance metrics related to classification models (except for the error rate), higher values indicate better performance.

The computational form of ICOMP-ROC and AIC-ROC for the ML classification algorithm is the same as in Equations (21) and (25), except, here, in scoring ROC curves, we use the results from the ML classification algorithms to compute the probability estimates since the actual response values and predicted response values are obtained. Then, these results are used in the standard ROC curve in the **perfcurve.m** function of MATLAB R2024a to compute the correlation matrix *R* to score ICOMP-ROC for each of the ML classification algorithms.

### 7.4. Main Computational Results

**Results of Example 1**: Table 10 presents the performance metrics and information-based model selection criteria for classification algorithms applied to the MRI Brain dataset.

All of our analysis was performed in MATLAB^®^ computational platform. Bold values indicate the best classifier according to performance metrics and model selection criteria.

According to Table 10, based on both performance metrics and information-based criteria, the best classification algorithm across full features, GA1, GA2, and GA1 ∩ GA2 is a Decision Tree (DT), while the worst-performing algorithm is Naive Bayes (NB). When the performance metrics are examined individually on full features a GA1, GA2, and GA1 ∩ GA2 datasets for DT classification algorithm, the full feature data perform best for accuracy (0.9520), recall (0.9726), F1 score (0.9617), and error rate (0.0480), while the GA1 leads in precision (0.9603) and GA2 performs best for AUC (0.9873). However, when considering information-based criteria, both AIC-ROC and ICOMP-ROC identify the GA1 as optimal, with DT achieving the lowest AIC-ROC (−74,696.0000) and ICOMP-ROC (−74,700.0000) values. In conclusion, for the Brain MRI dataset, the Decision Tree (DT) should be selected as the classification method due to its consistently superior performance across all metrics. While performance metrics do not clearly indicate a single best dataset, both AIC-ROC and ICOMP-ROC values consistently show that the GA1 is the most optimal among all the datasets. Therefore, the combination of DT with GA1 provides the most effective solution for this classification task.

**Table 10:** Comparison of performance metrics and model selection criteria for the Brain MRI dataset.

		Accuracy	Precision	Recall	F1Score	Error Rate	AUC	AIC-ROC	ICOMP-ROC
Full Features{1:17}	LR	0.7708	0.8012	0.8387	0.8195	0.2292	0.8269	−68,711.0000	−68,714.0000
	SVM	0.7768	0.8040	0.8468	0.8248	0.2232	0.8229	−5369.1000	−5371.7000
	NB	0.6467	0.7397	0.6645	0.7001	0.3534	0.7125	2891.6000	2890.0000
	KNN	0.8348	0.8331	0.9177	0.8734	0.1652	0.9050	−1419.9000	−1423.0000
	DT	**0.9520**	**0.9511**	**0.9726**	**0.9617**	**0.0480**	**0.9846**	**−71,927.0000 **	**−71,930.0000**
GA1{2,3,4,7,8,9,10,11,14,15,17}	LR	0.7508	0.7756	0.8419	0.8074	0.2493	0.7981	−68,281.0000	−68,284.0000
	SVM	0.7458	0.7699	0.8419	0.8043	0.2543	0.7952	−3815.6000	−3818.0000
	NB	0.6256	0.7584	0.5823	0.6588	0.3744	0.7155	4063.3000	4061.8000
	KNN	0.8459	0.8457	0.9194	0.8810	0.1542	0.9187	−189.6200	−192.8300
	DT	**0.9369**	**0.9603**	**0.9371**	**0.9486**	**0.0631**	**0.9857**	**−74,696.0000**	**−74,700.0000**
GA2{2,3,5,7,8,11,14,16}	LR	0.7568	0.7818	0.8436	0.8115	0.2432	0.8045	−63,835.0000	−63,837.0000
	SVM	0.7538	0.7750	0.8500	0.8108	0.2463	0.8008	−7741.9000	−7744.3000
	NB	0.5876	0.7921	0.4548	0.5779	0.4124	0.7154	4883.2000	4881.6000
	KNN	0.8348	0.8283	0.9258	0.8743	0.1652	0.9105	−965.5900	−968.7300
	DT	**0.9490**	**0.9523**	**0.9661**	**0.9592**	**0.0511**	**0.9873**	**−70,752.0000**	**−70,756.0000**
GA1 ∩ GA2{2,3,7,8,11,14}	LR	0.7017	0.7118	0.8726	0.7841	0.2983	0.7249	−63,753.0000	−63,754.0000
	SVM	0.7007	0.6999	0.9065	0.7899	0.2993	0.7253	−6817.0000	−6818.8000
	NB	0.6777	0.6915	0.8677	0.7697	0.3223	0.6992	171.2600	169.4400
	KNN	0.8078	0.8138	0.8952	0.8525	0.1922	0.8929	−1256.7000	−1259.7000
	DT	**0.9289**	**0.9449**	**0.9403**	**0.9426**	**0.0711**	**0.9816**	**−72,029.0000**	**−72,033.0000**

**Figure 8:**
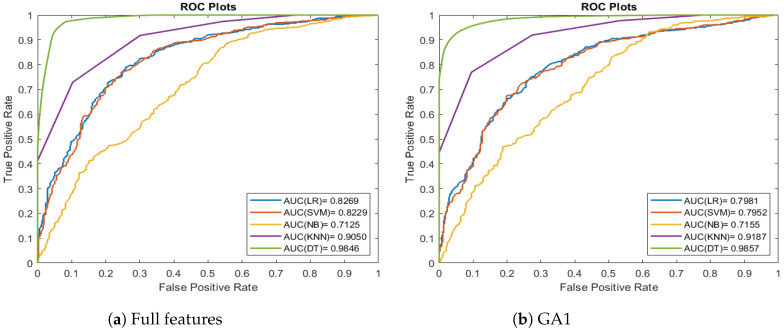

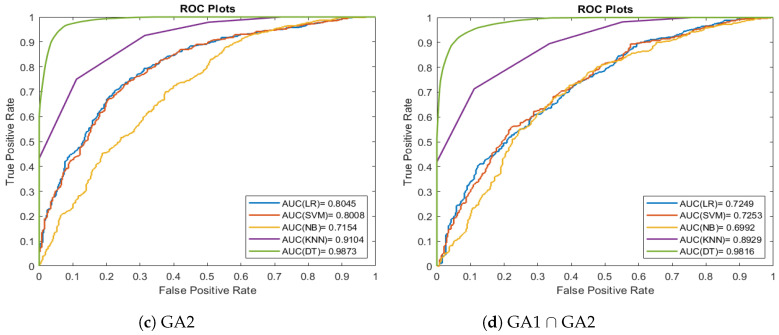
Comparison of classification methods ROC curves for the Brain MRI dataset.

Figure 8 presents the ROC curves for the classification algorithms applied to the Brain MRI datasets. In Figure 8, the ROC curves are clearly distinguishable, allowing for a straightforward comparison of the classifiers’ performance. Based on the AUC values, DT is the best-performing model across all datasets.

**Results of Example 2**: Table 11 presents the performance metrics and information-based model selection criteria for classification algorithms applied to the Wisconsin Breast Cancer datasets. Bold values indicate the best classifier according to performance metrics and model selection criteria.

According to Table 11, performance metrics (accuracy, F1 score, error rate), and information-based criteria (AIC-ROC and ICOMP-ROC), the best classification algorithm across the full features, GA1, GA2, and GA1 ∩ GA2 is Decision Tree (DT). Precision identifies logistic regression as the best classification algorithm for full features, recall highlights SVM as the best algorithm for the GA1, while AUC indicates KNN as the leading classifier for both full features and GA1. When the performance metrics are examined individually for full features, GA1, GA2 and GA1 ∩ GA2, the full features perform best for precision (0.9952) and recall (0.9953), while GA2 performs best for accuracy (0.9912), F1 score (0.9882), error rate (0.0088), AUC (0.9995), and information-based criteria AIC-ROC (−43,266.0000) and ICOMP-ROC (−43,270.0000) values. In conclusion, for the Wisconsin Breast Cancer dataset, considering all performance metrics except for precision and recall, and taking into account information-based criteria, Decision Tree (DT) combined with the GA2 provides the most effective solution for classification.

**Table 11:** Comparison of performance metrics and model selection criteria for the WBC dataset.

		Accuracy	Precision	Recall	F1Score	Error Rate	AUC	AIC-ROC	ICOMP-ROC
Full Features{1:30}	LR	0.9877	**0.9952**	0.9717	0.9833	0.0123	0.9845	−1205.1000	−1208.8000
	SVM	0.9877	0.9904	0.9764	0.9834	0.0123	0.9973	−2051.4000	−2054.9000
	NB	0.9403	0.9363	0.9009	0.9183	0.0598	0.9887	−737.5500	−741.2200
	KNN	0.9807	0.9951	0.9528	0.9735	0.0193	**0.9983**	−1251.6000	−1255.5000
	DT	**0.9895**	0.9769	**0.9953**	**0.9860**	**0.0105**	0.9978	**−40,899.0000**	**−40,903.0000**
GA1{1,2,3,5,7,9,13,17,19,20,22,25,27}	LR	0.9754	0.9714	0.9623	0.9668	0.0246	0.9950	−40,347.0000	−40,350.0000
	SVM	0.9789	0.9717	**0.9717**	0.9717	0.0211	0.9946	−2188.0000	−2191.5000
	NB	0.9473	0.9691	0.8868	0.9261	0.0527	0.9880	−98.4600	−101.9800
	KNN	0.9754	0.9714	0.9623	0.9668	0.0246	**0.9975**	−2040.4000	−2044.3000
	DT	**0.9842**	**0.9903**	0.9670	**0.9785**	**0.0158**	0.9972	**−41,051.0000**	**−41,055.0000**
GA2{1,3,5,8,11,14,16,17,18,20,22,26,27}	LR	0.9789	0.9808	0.9623	0.9714	0.0211	0.9974	−39,287.0000	−39,290.0000
	SVM	0.9789	0.9808	0.9623	0.9714	0.0211	0.9965	−3225.8000	−3229.3000
	NB	0.9315	0.9304	0.8821	0.9056	0.0685	0.9837	−416.0700	−419.5700
	KNN	0.9807	0.9809	0.9670	0.9739	0.0193	0.9977	−3290.6000	−3294.5000
	DT	**0.9912**	**0.9905**	**0.9859**	**0.9882**	**0.0088**	**0.9995**	**−43,266.0000**	**−43,270.0000**
GA1 ∩ GA2{1,3,5,17,20,22,27}	LR	0.9684	0.9664	0.9481	0.9571	0.0316	0.9901	−39,915.0000	−39,918.0000
	SVM	0.9649	0.9753	0.9293	0.9517	0.0351	0.9895	−3698.1000	−3701.6000
	NB	0.9262	0.9167	0.8821	0.8990	0.0738	0.9780	−2632.4000	−2635.9000
	KNN	0.9631	0.9614	0.9387	0.9499	0.0369	0.9944	−790.1600	−794.0500
	DT	**0.9789**	**0.9762**	**0.9670**	**0.9716**	**0.0211**	**0.9972**	**−41,227.0000**	**−41,230.0000**

**Figure 9:**
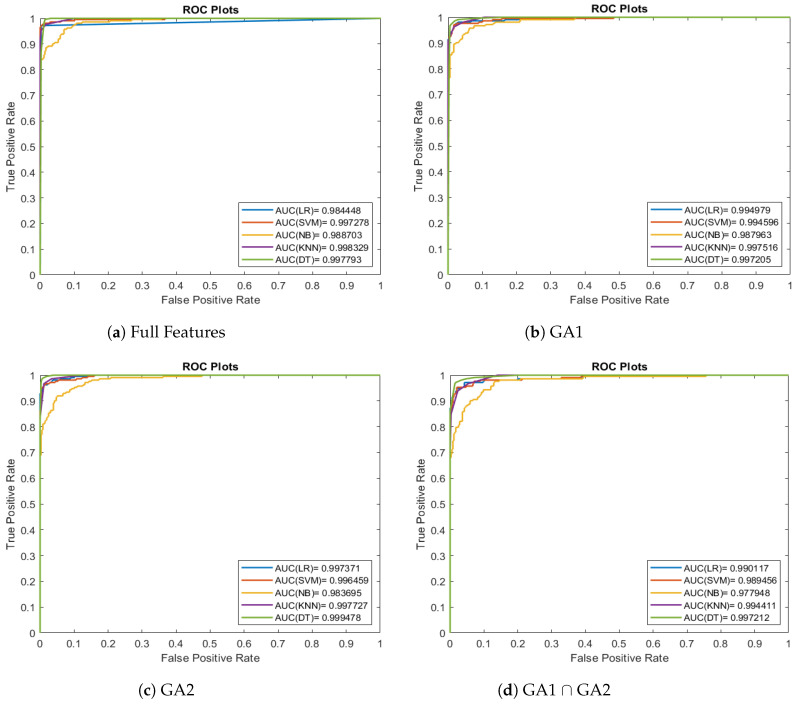
Comparison of classification methods ROC curves for Wisconsin Breast Cancer data set.

Figure 9 presents the ROC curves for the classification algorithms applied to the Wisconsin Breast Cancer datasets. In Figure 9, the ROC curves are too close; this indicates that the classifiers make it harder to differentiate between them based solely on the ROC curves.

## 8. Conclusions and Discussion

In this paper, we proposed and introduced new ICOMP-ROC and AIC-ROC information criteria for model selection to choose the best Bi-distributional ROC curve model among a portfolio of Bi-distributions. More specifically, we constructed sixteen different Bi-distribution combinations in a large-scale Monte Carlo simulation, and we empirically compared the performance of ICOMP-ROC and AIC-ROC criteria with the widely known AUC (Area Under the Curve) from the literature to choose the best-fitting Bi-distribution. Our simulation results are based on an imbalanced dataset. That is, we used different sample sizes for the negative class $\left(X_{1}\right)$ and positive class $\left(X_{2}\right)$. From Case 1 of our simulation experiment, our results showed that the pseudo true Normal–Normal distribution pair achieved a 100% success hit rate using ICOMP-ROC compared to AUC, which identified the GP-Normal distribution pair and AIC-ROC identified the Exponential–Exponential distribution pair as the best fit, with a 100% success rate, but failed to recognize the correct distribution as the Normal–Normal distribution pair. In addition, in Case 2 of our simulation experiment, we mixed the Weibull–Gamma distribution as a pair of pseudo-true distribution. In this scenario, our results showed that ICOMP-ROC and AIC-ROC criteria correctly identified the pseudo-true Weibull–Gamma distribution pair; in contrast, the AUC incorrectly identified the Weibull–Exponential pair. This is not surprising for the performance of the AUC, even though it is used as a popular metric in ROC curve modeling. As discussed in Halligan et al. [26], AUC has some drawbacks and limitations. The computational cost of AUC is high since AUC is inherently dependent upon the estimation of the ROC curve. The computational complexity of AUC is high for multiclass problems, as reported in Hand and Till [27], Provost and Domingos [28].

Although we relied on the simulation studies in choosing the best Bi-distributions in ROC curve modeling in the first part of the paper, our proposed approach can be easily applied to real dataset scenarios in which Bi-distributional fitting is required for the negative class $\left(X_{1}\right)$ and positive class $\left(X_{2}\right)$ .

Encouraged by the results of the Bi-distributional fitting, in the second part of the paper, we considered several well-known classification machine learning (ML) algorithms and studied their performance on real Magnetic Resonance Imaging (MRI) of the Brain data and Wisconsin Breast Cancer datasets. More specifically, we considered logistic regression (LR), support vector machines (SVMs), Naive Bayes (NB), k-nearest neighbor (KNN), and Decision Trees (DTs). We briefly provided an overview of these algorithms. There are other classification algorithms, which are listed in Section 2 on recent related work. Our purpose in this part of the paper was to understand how to select an optimal ML classification model. As is well known, there are many traditional performance evaluation measures when it comes to selecting a classification model. We studied the performance of newly proposed novel information-based criteria, namely, ICOMP-ROC and AIC-ROC, along with other traditional classification performance metrics. From the practical point of view, our goal was to choose a classification algorithm with the best predictive performance on the real datasets considered.

In all the ML classification algorithms, the information regarding the performance of these algorithms, as we discussed, is summarized in a confusion matrix. This matrix is built by comparing the observed and predicted classes for a set of observations. It contains all the information needed to calculate most of the traditional classification performance metrics such as accuracy, precision, recall, F1-score, error rate, and others.

While attempting to determine the true distribution pair for TPR and FPR, and to identify the best ML classification algorithm for real data, it was observed that performance metrics introduced challenges in the decision-making process. The inconsistencies and differing recommendations provided by each performance metric made it difficult to arrive at a clear conclusion regarding the optimal choice. However, we showed that ICOMP-ROC provides a clearer and more reliable assessment of classifier performance by effectively integrating both predictive accuracy and model complexity, thus supporting a more informed decision-making process in classifier selection. As noted in the literature, the basic key difference between the ICOMP criterion and AIC is ICOMP’s inclusion of complexity, which takes into account the correlation structure in the parameter estimates. Although in the classification results, ICOMP-ROC and AIC-ROC are minimized at the same ML classifier, we emphasize the fact that ICOMP-ROC, due to its ability to account for model complexity through the complexity of the celebrated inverse Fisher information matrix, provides a more robust criterion in high dimensions and eliminates counting and penalizing the number of parameters in the model explicitly.

Additionally, the use of genetic algorithms (GA) in this study plays a crucial role in optimizing the feature selection process. GA was employed to reduce the dimensionality of the datasets by identifying the most relevant features that contribute to classification accuracy. By mimicking the process of natural selection, GA iteratively refines the feature set, improving classification performance while reducing computational complexity. The usage of the GA is particularly important in this study as it helps minimize the model’s complexity, which is essential for more efficient and interpretable models, thereby helping to improve the accuracy of ML classification algorithms.

We are cognizant of the fact that in some complex data problems, GA can be slow, and it will need speeding up. For more on the theoretical and convergence properties of the GA, we refer the readers to Vose [29]. Further, we do not assume that ROC curve modeling is suitable for all datasets. The study of the impact of class imbalance on classification performance has been undertaken by Luque et al. [30]. Our approach can also handle the imbalanced data, as we illustrated in the Monte Carlo simulation study as well as in the real datasets.

There are other bivariate probability distributions to consider to score the ROC curve other than the bivariate normal (or Gaussian) distribution to guard against the non-Gaussianity in datasets. In future studies, we will consider other bivariate probability distributions along with relaxing the Bi-distributional assumption and study the performance of the nonparametric approach to ROC curve modeling using the kernel density estimation (KDE) approach. Further, we will generalize binary classification results to multi-class classification problems when we have more than two groups.

## Figures and Tables

**Table 1:** Probability distributions.

Distributions	pdf
Normal	$f\left(x|\mu ,\sigma \right)=\frac{1}{\sigma \sqrt{2\pi }}exp\left\{-\frac{{\left(x-\mu \right)}^{2}}{2\sigma^{2}}\right\}$, for x∈R
Exponential	$f\left(x|\mu \right)=\frac{1}{\mu }e^{-\frac{x}{\mu }}$
Weibull	$f\left(x|\alpha ,\beta \right)=\frac{\alpha }{\beta^{\alpha }}x^{\alpha -1}e^{-{\left(\frac{x}{\beta }\right)}^{\alpha }}$, x>0, α, β>0
Gamma	$f\left(x|\alpha ,\beta \right)=\frac{\beta^{\alpha }}{\Gamma \left(\alpha \right)}x^{\alpha -1}e^{-\beta x}$, x>0, α, β>0
EV	$f\left(x|\mu ,\sigma \right)=\left(\frac{1}{\sigma }\right)e^{\left(\frac{x-\mu }{\sigma }\right)}\exp\left(-e^{\left(\frac{x-\mu }{\sigma }\right)}\right)$
GEV	$f\left(x|\mu ,\sigma ,k\right)=\left(\frac{1}{\sigma }\right)g\left(x\right)e^{-g\left(x\right)}$,
	where $g\left(x\right)={\left[1+k\left(\frac{x-\mu }{\sigma }\right)\right]}^{-1/k}$ if k≠0
GP	$f\left(x|\mu ,\sigma ,k\right)=\left(\frac{1}{\sigma }\right){\left(1+\frac{k\left(x-\mu \right)}{\sigma }\right)}^{\left(-\frac{1}{k}-1\right)}$
	for x≥μ when k≥0, and μ≤x≤μ−σ/k when k<0

**Table 2:** Cumulative distributions functions.

Distributions	cdf
Normal	$F\left(x|\mu ,\sigma \right)=\frac{1}{\sigma \sqrt{2\pi }}\int_{-\infty }^{x}e^{-{\left(\frac{t-\mu }{}\right)}^{2}}dt$
Exponential	$F\left(x|\mu \right)=1-e^{-\frac{x}{\mu }}$
Weibull	$F\left(x|\alpha ,\beta \right)=1-e^{-{\left(\frac{x}{\beta }\right)}^{\alpha }}$
Gamma	$F\left(x|\alpha ,\beta \right)=\frac{I\left(\alpha ,\beta x\right)}{\Gamma \left(\alpha \right)}$,
	$I\left(\alpha ,\beta x\right)$ = incomplete gamma function
EV	$F\left(x|\mu ,\sigma \right)=1-exp\left(-e^{\left(\frac{x-\mu }{\sigma }\right)}\right)$
GEV	$F\left(x|\mu ,\sigma ,k\right)=e^{-g\left(x\right)}$
GP	$F\left(x|\mu ,\sigma ,k\right)=1-{\left(1+\frac{k\left(x-\mu \right)}{\sigma }\right)}^{-1/k}$ for k≠0

**Table 3:** Inverse cumulative distributions functions.

Distributions	icdf
Normal	$x_{p}=F^{-1}\left(p|\mu ,\sigma \right)=\left\{x:F(x|\mu ,\sigma )=p\right\}$
	where$\;p=F\left(x|\mu ,\sigma \right)=\frac{1}{\sigma \sqrt{2\pi }}\int_{-\infty }^{x}e^{-{\left(\frac{t-\mu }{}\right)}^{2}}dt$
Exponential	$x_{p}=F^{-1}\left(p|\mu \right)=-\mu log\left(1-p\right)$
Weibull	$x_{p}=F^{-1}\left(p|\alpha ,\beta \right)=-\alpha {\left[log\left(1-p\right)\right]}^{1/\beta }$
Gamma	$x_{p}=F^{-1}\left(p|\alpha ,\beta \right)=\left\{x:F\left(x\right)=p\right\}$
	where $p=F\left(x|\alpha ,\beta \right)=\frac{1}{\beta^{\alpha }\Gamma \left(\alpha \right)}\int_{0}^{x}t^{\alpha -1}e^{-\frac{t}{\beta }}dt$
EV	$x_{p}=F^{-1}\left(p|\mu ,\sigma \right)$
GEV	$x_{p}=F^{-1}\left(p|\mu ,\sigma ,k\right)=\mu +\frac{\sigma }{k}\left(\left(-log{\left(p\right)}^{-k}-1\right)\right)$ for k>0
GP	$x_{p}=F^{-1}\left(p|\mu ,\sigma ,k\right)$

**Table 4:** Results obtained by running the Monte Carlo simulation once for the Normal–Normal Bi-distribution pair.

Positive Class	Negative Class	AUC	AIC-ROC	ICOMP-ROC
Normal (120.25, 10.88)	Normal (121.44, 9.91)	0.5322	924.2721	**926.1975**
Exponential (120.25, 0)	Exponential (121.44, 0)	0.5025	**922.9439**	930.3891
Weibull (125.28, 11.03)	Weibull (125.94, 13.37)	0.5289	925.1229	926.5540
Gamma (123.19, 0.98)	Gamma (147.58, 0.82)	0.5335	924.3183	926.2096
EV (125.81, 11.71)	EV (126.31, 9.47)	0.5261	925.2603	926.6303
GEV (116.01, 10.43)	GEV (118.06, 10.18)	0.5404	926.7968	927.6573
GP (−1.43, 222.86)	GP (−1.6, 236.08)	0.4863	925.4887	926.7646
Exponential (120.25, 0)	Normal (121.44, 9.91)	0.6345	1036.5695	1033.9353
Gamma (123.19, 0.98)	Normal (121.44, 9.91)	0.5370	925.3379	926.6749
Weibull (125.28, 11.03)	Normal (121.44, 9.91)	0.5210	925.5925	926.8284
EV (125.81, 11.71)	Normal (121.44, 9.91)	0.5177	927.6919	928.3429
GEV (116.01, 10.43)	Normal (121.44, 9.91)	0.5385	926.6435	927.5447
GP (−1.43, 222.86)	Normal (121.44, 9.91)	**0.6558**	1019.5691	1017.1146
Weibull (125.28, 11.03)	Gamma (147.58, 0.82)	0.4817	926.0646	927.1363
Exponential (125.28, 11.03)	Exponential (121.44, 0)	0.6245	1027.2664	1024.7273
Exponential (123.19, 0.98)	Exponential (121.44, 0)	0.6270	1032.3725	1029.7804

Note: Normal (μ, σ) with mean μ and standard deviation σ; Exponential (λ) with rate parameter λ; Weibull (α, β) with scale β, and shape α; Gamma (α, β) with scale β, and shape α; Extreme Value (EV) (μ, σ) with mean μ and standard deviation σ; Generalized Extreme Value (GEV) (*k*, σ, μ) with location parameter μ, scale parameter σ, and shape parameter *k*; Generalized Pareto (GP) (*k*, σ) with shape parameter *k* and, scale parameter σ.

**Table 5:** Frequency of success for the Monte Carlo simulation study for the Normal–Normal Bi-distribution pair.

Positive Class	Negative Class	AUC	AIC-ROC	ICOMP-ROC
Normal (1, 1.87)	Normal (0.99, 0.71)	0	0	**100**
Exponential (1.98)	Exponential (0.99)	0	**100**	0
Weibull (2.00, 1.02)	Weibull (1.10, 1.47)	0	0	0
Gamma (1.03, 1.91)	Gamma (1.98, 0.50)	0	0	0
EV (3.03, 2.54)	EV (1.39, 0.93)	0	0	0
GEV (0.49, 0.92, 0.90)	GEV (0.16, 0.46, 0.64)	0	0	0
GP (−0.07, 2.13)	GP (−0.25, 1.23)	0	0	0
Exponential (1.98)	Normal (0.99, 0.71)	0	0	0
Gamma (1.03, 1.91)	Normal (0.99, 0.71)	0	0	0
Weibull (2.00, 1.02)	Normal (0.99, 0.71)	0	0	0
EV (3.03, 2.54)	Normal (0.99, 0.71)	0	0	0
GEV (0.49, 0.92, 0.90)	Normal (0.99, 0.71)	0	0	0
GP (−0.07, 2.13)	Normal (0.99, 0.71)	**100**	0	0
Weibull (2.00, 1.02)	Gamma (1.98, 0.50)	0	0	0
Weibull (2.00, 1.02)	Exponential (0.99)	0	0	0
Gamma (1.03, 1.91)	Exponential (0.99)	0	0	0

Note: Normal (μ, σ) with mean μ and standard deviation σ; Exponential (λ) with rate parameter λ; Weibull (α, β) with scale β, and shape α; Gamma (α, β) with scale β, and shape α; EV (μ, σ) with mean μ and standard deviation σ; GEV (*k*, σ, μ) with location parameter μ, scale parameter σ, and shape parameter *k*; GP (*k*, σ) with shape parameter *k* and, scale parameter σ.

**Table 6:** Results obtained by running the Monte Carlo simulation once for the Weibull–Gamma Bi-distribution pair.

Positive Class	Negative Class	AUC	AIC-ROC	ICOMP-ROC
Normal (1.98, 1.87)	Normal (0.99, 0.71)	0.3106	1003.7150	1001.4560
Exponential (1.98)	Exponential (0.99)	0.3338	953.0782	951.8567
Weibull (2.00, 1.02)	Weibull (1.10, 1.47)	0.3542	957.7638	956.3931
Gamma (1.03, 1.91)	Gamma (1.98, 0.50)	0.3542	960.5215	959.0718
EV (3.03, 2.54)	EV (1.39, 0.93)	0.3620	988.2994	986.2680
GEV (0.49, 0.92, 0.90)	GEV (0.16, 0.46, 0.64)	0.3579	955.4247	954.1259
GP (−0.07, 2.13)	GP (−0.25, 1.23)	0.3340	952.7373	951.5275
Exponential (1.98)	Normal (0.99, 0.71)	0.3693	951.8894	950.7093
Gamma (1.03, 1.91)	Normal (0.99, 0.71)	0.3641	952.3061	951.1113
Weibull (2.00, 1.02)	Normal (0.99, 0.71)	0.3624	952.7872	951.5757
EV (3.03, 2.54)	Normal (0.99, 0.71)	0.3676	1006.6130	1004.3160
GEV (0.49, 0.92, 0.90)	Normal (0.99, 0.71)	0.3774	941.7358	940.9978
GP (−0.07, 2.13)	Normal (0.99, 0.71)	0.3571	955.0989	953.8105
Weibull (2.00, 1.02)	Gamma (1.98, 0.50)	0.6475	**938.0450**	**937.5293**
Weibull (2.00, 1.02)	Exponential (0.99)	**0.6728**	944.7688	943.8782
Gamma (1.03, 1.91)	Exponential (0.99)	0.6715	944.0736	943.2161

Note: Normal (μ, σ) with mean μ and standard deviation σ; Exponential (λ) with rate parameter λ; Weibull (α, β) with scale β, and shape α; Gamma (α, β) with scale β, and shape α; Extreme Value (EV) (μ, σ) with mean μ and standard deviation σ; Generalized Extreme Value (GEV) (*k*, σ, μ) with location parameter μ, scale parameter σ, and shape parameter *k*; Generalized Pareto (GP) (*k*, σ) with shape parameter *k* and, scale parameter σ.

**Table 7:** Frequency of success for the Monte Carlo simulation study for the Weibull–Gamma Bi-distribution pair.

Positive Class	Negative Class	AUC	AIC-ROC	ICOMP-ROC
Normal (1.98, 1.87)	Normal (0.99, 0.71)	0	0	0
Exponential (1.98)	Exponential (0.99)	0	0	0
Weibull (2.00, 1.02)	Weibull (1.10, 1.47)	0	0	0
Gamma (1.03, 1.91)	Gamma (1.98, 0.50)	0	0	0
EV (3.03, 2.54)	EV (1.39, 0.93)	0	0	0
GEV (0.49, 0.92, 0.90)	GEV (0.16, 0.46, 0.64)	0	0	0
GP (−0.07, 2.13)	GP (−0.25, 1.23)	0	0	0
Exponential (1.98)	Normal (0.99, 0.71)	0	0	0
Gamma (1.03, 1.91)	Normal (0.99, 0.71)	0	0	0
Weibull (2.00, 1.02)	Normal (0.99, 0.71)	0	0	0
EV (3.03, 2.54)	Normal (0.99, 0.71)	0	0	0
GEV (0.49, 0.92, 0.90)	Normal (0.99, 0.71)	0	0	0
GP (−0.07, 2.13)	Normal (0.99, 0.71)	0	0	0
Weibull (2.00, 1.02)	Gamma (1.98, 0.50)	0	**100**	**100**
Weibull (2.00, 1.02)	Exponential (0.99)	**100**	0	0
Gamma (1.03, 1.91)	Exponential (0.99)	0	0	0

Note: Normal (μ, σ) with mean μ and standard deviation σ; Exponential (λ) with rate parameter λ; Weibull (α, β) with scale β, and shape α; Gamma (α, β) with scale β, and shape α; EV (μ, σ) with mean μ and standard deviation σ; GEV (*k*, σ, μ) with location parameter μ, scale parameter σ, and shape parameter *k*; GP (*k*, σ) with shape parameter *k* and, scale parameter σ.

**Table 8:** GA parameters.

	GA1	GA2
Number generations	50	50
Population size	100	100
Crossover probability	0.8	0.6
Mutation probability	0.3	0.001

**Table 9:** Performance metrics.

Performance Metrics	Definition	
Accuracy	$\frac{TP+TN}{TP+FN+FP+TN}$	(35)
Precision	$\frac{TP}{TP+FP}$	(36)
Recall	$\frac{TP}{TP+FN}$	(37)
F1 Score	$\frac{2\times (Recall\times Precision)}{Recall+Precision}\;$	(38)
Error Rate	$\frac{FP+FN}{TP+FN+FP+TN}$	(39)

## Data Availability

MRI image of the brain and Wisconsin Breast Cancer datasets used in this paper can be downloaded from source: https://www.kaggle.com/datasets/navoneel/brain-mri-images-for-brain-tumor-detection (accessed on 1 June 2024), and https://www.kaggle.com/datasets/uciml/breast-cancer-wisconsin-data (accessed on 10 October 2024), respectively.

## Abbreviations

The following abbreviations are used in this manuscript:

CTT	Classic test theory	CAIC	Consistent AIC
MSA	Measurement System Analysis	CAICF	Consistent AIC with Fisher information
IRT	Item Response Theory	ICOMP	Information Complexity
WBC	Wisconsin Breast Cancer	ICOMP-ROC	Information Complexity-ROC
BC	Breast Cancer	IFIM	Inverse-Fisher information matrix
EML	Ensemble Machine Learning	BSB1	Bivariate Selberg-beta type 1
RFE	Recursive Feature Elimination	MLE	Maximum likelihood estimate
ML	Machine Learning	MRI	Magnetic Resonance Imaging
GA	Genetic Algorithm	GLCM	Gray level co-occurance matrix
NN	Neural Network	TP	True positive
PV	Photovoltaic	FN	False negative
RNN	Recurrent Neural Network	FP	False positive
LSTM	Long Short-Term Memory	TN	True negative
CNN	Convolution Neural Network	TPR	True Positive Rate
FFNN	Feed-Forward Neural Network	FPR	False Positive Rate
CFNN	Cascade-Forward Neural Network	LR	Logistic regression
IWO	Invasive Weed Optimization	SVM	Support vector machine
AUC	Area under curve	NB	Naive Bayes
UROC	Universal ROC	KNN	K-nearest neighbor
AIC	Akaike’s information criterion	DT	Decision Tree
ROC	Receiver Operating Characteristic	TIC	Takeuchi information criterion
GKQ	Gaus-Kronrod Quadrature	EV	Extreme Value
GEV	Generalized Extreme Value	GP	Generalized Pareto
KDE	Kernel Density Estimation	CDF	Cumulative Distribution Function
PDF	Probability Density Function		
